# Eye movements in patients in early psychosis with and without a history of cannabis use

**DOI:** 10.1038/s41537-021-00155-2

**Published:** 2021-05-12

**Authors:** Musa Basseer Sami, Luciano Annibale, Aisling O’Neill, Tracy Collier, Chidimma Onyejiaka, Savitha Eranti, Debasis Das, Marlene Kelbrick, Philip McGuire, Steve C. R. Williams, Anas Rana, Ulrich Ettinger, Sagnik Bhattacharyya

**Affiliations:** 1 Institute of Psychiatry, Psychology and Neurosciences King’s College London, London, UK; 2 Institute of Mental Health, Nottingham University, Nottingham, England; 3 East London Foundation Trust, London, UK; 4 Leicestershire Partnership NHS Trust, Leicester, UK; 5 Northamptonshire Healthcare NHS Foundation Trust, Kettering, UK; 6 Centre for Neuroimaging Sciences, King’s College London, London, UK; 7 Centre for Computational Biology, University of Birmingham, Birmingham, UK; 8 Department of Psychology, University of Bonn, Bonn, Germany

**Keywords:** Biomarkers, Psychosis

## Abstract

It is unclear whether early psychosis in the context of cannabis use is different from psychosis without cannabis. We investigated this issue by examining whether abnormalities in oculomotor control differ between patients with psychosis with and without a history of cannabis use. We studied four groups: patients in the early phase of psychosis with a history of cannabis use (EPC; *n* = 28); patients in the early phase of psychosis without (EPNC; *n* = 25); controls with a history of cannabis use (HCC; *n* = 16); and controls without (HCNC; *n* = 22). We studied smooth pursuit eye movements using a stimulus with sinusoidal waveform at three target frequencies (0.2, 0.4 and 0.6 Hz). Participants also performed 40 antisaccade trials. There were no differences between the EPC and EPNC groups in diagnosis, symptom severity or level of functioning. We found evidence for a cannabis effect (*χ*^2^ = 23.14, *p* < 0.001), patient effect (*χ*^2^ = 4.84, *p* = 0.028) and patient × cannabis effect (*χ*^2^ = 4.20, *p* = 0.04) for smooth pursuit velocity gain. There was a large difference between EPC and EPNC (*g* = 0.76–0.86) with impairment in the non cannabis using group. We found no significant effect for antisaccade error whereas patients had fewer valid trials compared to controls. These data indicate that impairment of smooth pursuit in psychosis is more severe in patients without a history of cannabis use. This is consistent with the notion that the severity of neurobiological alterations in psychosis is lower in patients whose illness developed in the context of cannabis use.

## Introduction

A history of cannabis use is arguably one of the most widely implicated modifiable risk factors in early psychosis, with a third of patients using the drug regularly at first presentation^1^. Cannabis is a ‘component cause’ of psychotic disorder^2–4^, neither necessary nor sufficient for psychotic disorder but implicated in the causal pathway. Clinical practice algorithms advise reduction in use but do not categorically distinguish between patients who have used cannabis and who have not^5,6^. Neurobiological measures may provide evidence to support whether there is a biological distinction between patients who use and do not use psychosis.

Patients with early psychosis with a history of cannabis use (EPC) have a worse prognosis, unlikely to be a result of other drug use, poor adherence to treatment, genetic or environmental confounding or self-medication with cannabis in those with poor prognosis^7^. They spend an extra 35 days in hospital in the first 5 years of illness compared to patients with early psychosis without (EPNC)^8^, and over the course of 34 years this extends to an additional year of hospitalisation^9^. Consequently, cannabis-use or non-use may be a relevant demarcating factor in early psychosis in this heterogeneous clinical population.

The pathway to psychotic disorder has been conceptualised as an aggregation of interacting neurodevelopmental and other insults to meet the threshold for disorder^10,11^. Such insults may include a variety of risk factors implicated in the pathway to psychosis experienced throughout childhood and adolescence including genetic and familial risk, birth trauma, winter birth and childhood trauma^12–14^.

**Fig. 1:**
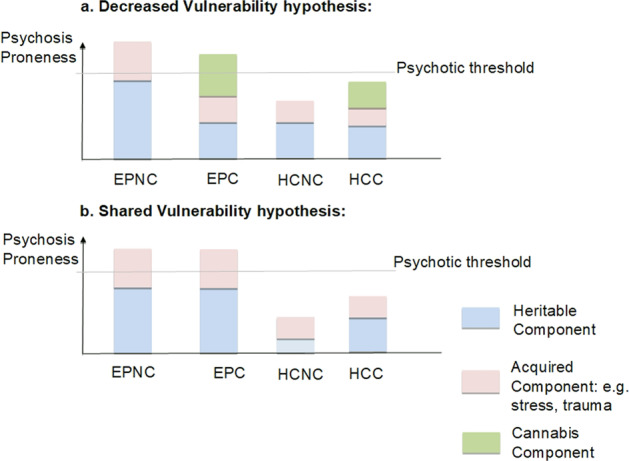
Conceptual models for understanding cannabis-psychosis vulnerability. **a** In the decreased vulnerability model, cannabis represents a distinct risk factor and less heritable predisposition to schizophrenia is required to reach the psychotic threshold. If oculomotor deficits represent a heritable endophenotype for schizophrenia, we would expect impairment in EPNC vs EPC. **b** In the shared vulnerability model there is no difference in heritable component of EPC vs EPNC and we would not expect to see any oculomotor difference in EPC vs EPNC. EPC early psychosis with cannabis use, EPNC early psychosis without cannabis use, HCC healthy controls with cannabis use, HCNC healthy controls without cannabis use, SPEM smooth pursuit eye movements, AS antisaccades.

Two alternative models of psychosis are shown in Fig. 1. In a model where a history of heavy cannabis use is an additional such insult, cannabis users may have a lower biological diathesis to psychosis compared to non-cannabis users (decreased vulnerability hypothesis)^15^. Consequently, there would be cases in the EPC group who would not have met the threshold for psychotic disorder without cannabis use. Accordingly, in the non-using EPNC group, there should be a stronger expression of markers of biological predisposition. Conversely it has been argued that the cannabis-psychosis association is explained by those prone to psychosis being also prone to cannabis use (shared vulnerability hypothesis)^16–18^. In that case one would not expect to see any difference in such markers in EPC than EPNC or, if cannabis use indexes increased pre-existing vulnerability, may see that chronic cannabis using patients display worse performance in such measures than patients without cannabis use.

One way of testing these competing hypotheses (decreased vulnerability vs. shared vulnerability) in a cross-sectional study is to test between groups for endophenotypes of psychotic disorder, which are known to be trait markers of the disorder and cosegregate with genetic risk. Oculomotor abnormalities are among the most widely replicated and reliable findings in psychotic disorders^19–21^ and have been demonstrated to differentiate patients with schizophrenia from controls with exceptional accuracy^22^. Smooth pursuit eye movement (SPEM) dysfunction, i.e., a deficit in following a slowly moving target, is considered to be a reliable endophenotype of schizophrenia^23,24^ and is a particularly attractive paradigm for the following reasons: (i) SPEM abnormalities are almost invariably present in patient groups compared to controls^24^, (ii) abnormalities are observed in relatives of patients indicating a degree of heritability^20^, (iii) abnormalities do not appear to be related to symptom load and may be considered a trait marker^25,26^. Similarly, antisaccade (AS) abnormalities, i.e., a deficit in making a rapid eye movement away from a sudden-onset stimulus^27^, have been demonstrated to be an endophenotype in psychotic disorders^28–30^ with abnormalities present from first presentation^31^ and a large trait component^32^.

Any such measure should ideally be relatively insensitive to the effects of cannabis outside of intoxication, to be used as markers of biological diathesis outside of cannabis use. In healthy individuals, SPEM appears less sensitive to chronic cannabis use than AS. Cannabis or delta-9-tetrahydrocannabinol (THC) has been shown to decrease performance in smooth pursuit acutely in some^33,34^ but not all studies^35–37^; however, these effects do not persist beyond acute intoxication^33^. Substance misuse is not associated with deterioration in smooth pursuit performance as seen in patients with schizophrenia^38^. Acute administration of THC has been shown to increase antisaccade error rate and latency^39^ whereas both anti and prosaccade latency have been shown to be increased in chronic cannabis users outside the intoxication state compared to non-using controls^40^.

One previous study showed alterations in visual scan paths in cannabis-induced psychotic disorder as distinct from patients with schizophrenia (including cannabis users) using data viewing landscapes in a small sample^41^. However, no study to date has aimed to differentiate cannabis-using from non-using patients using the well-established SPEM or AS markers of schizophrenia. In fact, many studies specifically excluded individuals with comorbid substance use^30,31,42,43^. Differences in eye movement measures between groups would indicate altered neurobiology between cannabis-using and non-using patients and may suggest a different degree of biological diathesis in the groups.

We, therefore, aimed to determine whether early psychosis patients with a history of cannabis and those without differ in in SPEM and AS performance. As primary outcomes we tested measures robustly associated with abnormalities in oculomotor control in psychosis: velocity gain to index SPEM^24^ and error rate to index AS^44^.

## Results

Baseline parameters are shown in Table 1. There were no significant differences between EPC and EPNC in clinical measures: time since diagnosis, age at diagnosis, current medication, days in hospital, PANSS scores and subscales, GAF scores and Social and General functioning (all *p* > 0.175). As expected, estimated full-scale IQ was lower in patients than controls, but there was no difference between EPC and EPNC. The only significant differences between EPC and EPNC were related to substance use: as expected, participants with EPC had higher indices of cannabis use and also scored higher on Fagerstrom and AUDIT scores.

**Table 1:** Demographic and clinical parameters by group.

	EPC			EPNC			HCC			HCNC		
	x̄/propn	SD	*n*	x̄/propn	SD	*n*	x̄/propn	SD	*n*	x̄/propn	SD	*n*
Male sex (proportion)	0.79	–	28	0.64	–	25	0.63	–	16	0.45	–	22
Age (x̄)	25.59	3.98	28	26.7	4.83	25	27.11	5.95	16	27.99	5.34	22
White (proportion)	0.46	–	28	0.32		25	0.44	–	16	0.36	–	22
Fagerstrom test of nicotine dependence (x̄)^a^^b^	2.39	2.38	28	0.52	1.26	25	0.75	1.73	16	0	0	22
AUDIT (x̄)^a^^b^	8.86	5.38	28	3.52	5.1	25	7.75	6.43	16	3.86	3.11	22
Estimated full scale intelligence quotient (x̄)	102.28	10.38	25	102.54	9.24	22	110.56	6.85	16	110.46	7.86	18
Affective psychosis diagnosis (proportion)	0.43	–	28	0.4	–	25	–	–	–	–	–	–
Schizophrenia spectrum disorder diagnosis (proportion)	0.75	–	28	0.84	–	25	–	–	–	–	–	–
Days spent in hospital (x̄)	51.79	75.94	28	54.68	70.62	25	–	–	–	–	–	–
Chlorpromazine equivalents (x̄)	182.77	165.06	28	192.9	177.11	25	–	–	–	–	–	–
Age of psychosis onset (x̄)	23.56	3.95	28	23.92	5.63	25	–	–	–	–	–	–
Months since illness onset (x̄)	23.72	15.53	28	36.86	48.31	25	–	–	–	–	–	–
PANSS – positive (x̄)	12.39	5.53	28	11.4	5.5	25	–	–	–	–	–	–
PANSS – negative (x̄)	14.11	7.29	28	14.4	6.87	25	–	–	–	–	–	–
PANSS – general (x̄)	27.75	9.2	28	26.16	8.7	25	–	–	–	–	–	–
PANSS – total (x̄)	54.25	18.52	28	51.96	17.82	25	–	–	–	–	–	–
GAF (x̄)	70.39	8.71	28	72.76	10.85	25	–	–	–	–	–	–
Social Functioning Score (x̄)^b^	7.09	1.19	22	7.25	1.62	20	8.57	0.94	14	9	0.32	20
General Functioning Score (x̄)^b^	6.68	0.89	22	6.95	1.47	20	8.64	0.93	14	8.9	0.55	20
Age first tried cannabis (x̄)	16.15	2.49	27	17.82	3.25	11	16	2.5	16	19.67	4.42	12
Days since last joint (x̄)	487.31	1115.39	28	–	–	–	769.63	1389.07	16	–	–	–
Cannabis use in last week (proportion)^a^^b^	0.32	–	28	0	–	25	0.5	–	16	0	–	22
Cannabis in urine drug sample (proportion)^ab^	0.43	–	28	0	–	25	0.5	–	16	0	–	22
Time to smoke 3.5g of cannabis (days) (x̄)	10.22	11.32	23	–	–		8.17	9.13	12	–	–	–
Money spent on cannabis in a week (£) (x̄)^ab^	30.21	26.33	28	0	–	25	35.63	38.16	16	0	–	22
Cannabis Severity of Dependence Scale (x̄)^ab^	1.54	2.72	28	0	–	25	1.87	2.42	16	0	–	22
Lifetime cannabis misuse diagnosis (proportion)^ab^	0.79	–	28	0	–	25	0.5	–	16	0	–	22
Current cannabis misuse diagnosis (proportion)^ab^	0.29	–	28	0	–	25	0.25	–	16	0	–	22
Lifetime alcohol misuse diagnosis (proportion)	0.14	–	28	0.12	–	25	0.19	–	16	0	–	22
Current alcohol misuse diagnosis (proportion)	0	–	28	0	–	25	0	–	16	0	–	22
Lifetime other drug misuse diagnosis (proportion)	0.07	–	28	0	–	25	0.06	–	16	0	–	22
Current other drug misuse diagnosis (proportion)	0	–	28	0	–	25	0	–	16	0	–	22

First column for each group reports mean where SD is specified, otherwise reports proportion. *T*-tests and *χ*^2^ between groups to test significance (Fisher’s exact test where numbers in cell < 5).

*EPC* early psychosis with cannabis use, *EPNC* early psychosis without cannabis use, *HCC* healthy controls with cannabis use, *HCNC* healthy controls without cannabis use, *SD* standard deviation, *x̄* mean, *propn* proportion, *PANSS* Positive and Negative Syndrome Scale, *GAF* global assessment of functioning.

^a^Significant difference between EPC vs EPNC (*t*-test, *χ*^2^).

^b^Significant group differences (ANOVA, *χ*^2^).

**Table 2:** Oculomotor measures by group.

	EPC	EPNC	HCC	HCNC
SPEM 0.2 Hz Gain	86.0 (7.1)	75.2 (16.6)	88.1 (5.0)	83.1 (7.6)
SPEM 0.4 Hz Gain	77.4 (12.9)	63.7 (21.8)	77.3 (8.4)	73.5 (14.3)
SPEM 0.6 Hz Gain	68.0 (12.9)	52.9 (25.0)	63.1 (18.5)	61.4 (18.7)
AS error rate (%)	33.3 (20.2)	35.2 (23.1)	29.7 (13.9)	24.8 (24.0)
AS valid trials (/40)	33.8 (4.1)	32.7 (8.3)	36.25 (3.6)	36.09 (4.2)

*EPC* early psychosis with cannabis use, *EPNC e*arly psychosis without cannabis use, *HCC* healthy controls with cannabis use, *HCNC* healthy controls without cannabis use, *SPEM s*mooth pursuit eye movements, *AS* antisaccades.

### SPEM velocity gain

**Fig. 2:**
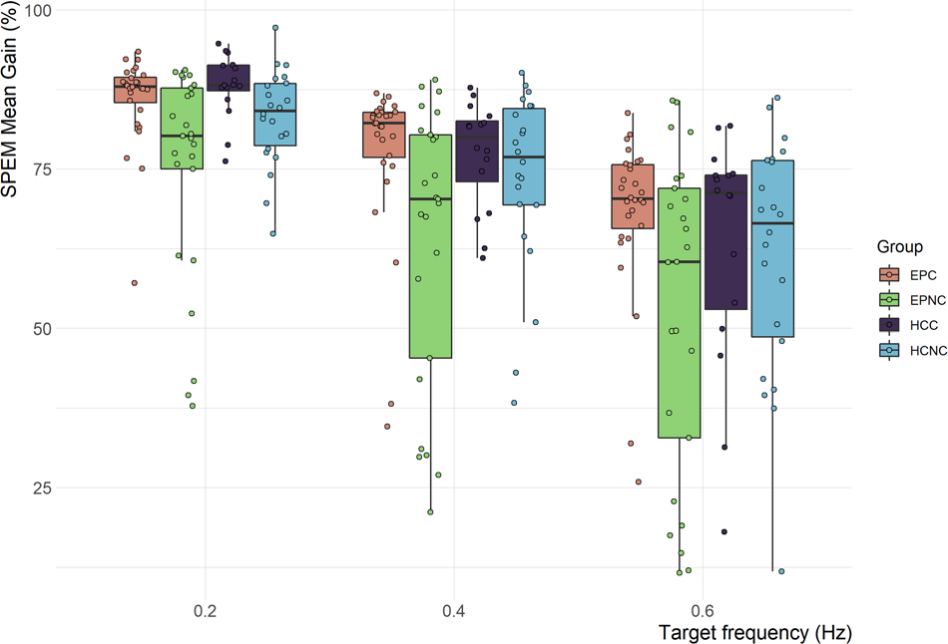
SPEM mean gain × group. EPC early psychosis with cannabis use, EPNC early psychosis without cannabis use, HCC healthy controls with cannabis use, HCNC healthy controls without cannabis use, SPEM smooth pursuit eye movements. Boxplot plotted using *ggplot geom_boxplot* defaults in *R*: centre lines represent median, box hinges indicate Quartile 1 & 3, whisker 1.5x Interquartile Range from hinge or furthest data point, whichever is closer.

Mean SPEM velocity gain by patient status, cannabis user status and stimulus frequency are shown in Fig. 2 and Table 2, indicating lower mean gain and increased variance with increasing stimulus frequency, as expected. Figure 2 also visualises that EPNC had lower mean velocity gain and larger variance compared to other groups. A full mixed model showed patients had lower mean velocity gain (*t* = −2.21) than controls and cannabis users had higher velocity mean gain (*t* = 4.91) than non-users. Testing of alternative models versus the full model showed evidence for a cannabis effect (*χ*^2^(1) = 23.14, *p* < 0.001), patient effect (*χ*^2^(1) = 4.84, *p* = 0.028) and patient × cannabis interaction effect (*χ*^2^(1) = 4.20, *p* = 0.04). There was no evidence for additional information from including AUDIT and Fagerstrom scores in the model (*χ*^2^(2) = 4.19, *p* = 0.063). To specifically determine the magnitude of difference in EPC vs EPNC, Hedge’s *g* showed impairment of EPNC of large effects of 0.86, 0.76 and 0.77 at 0.2, 0.4 and 0.6 Hz, respectively. Sensitivity analysis in a more restricted ‘ideal world’ sample to excluding comorbidity, learning disability and cigarette smoking 40 min before the test made no difference to these results.

### Antisaccade error rate

**Fig. 3:**
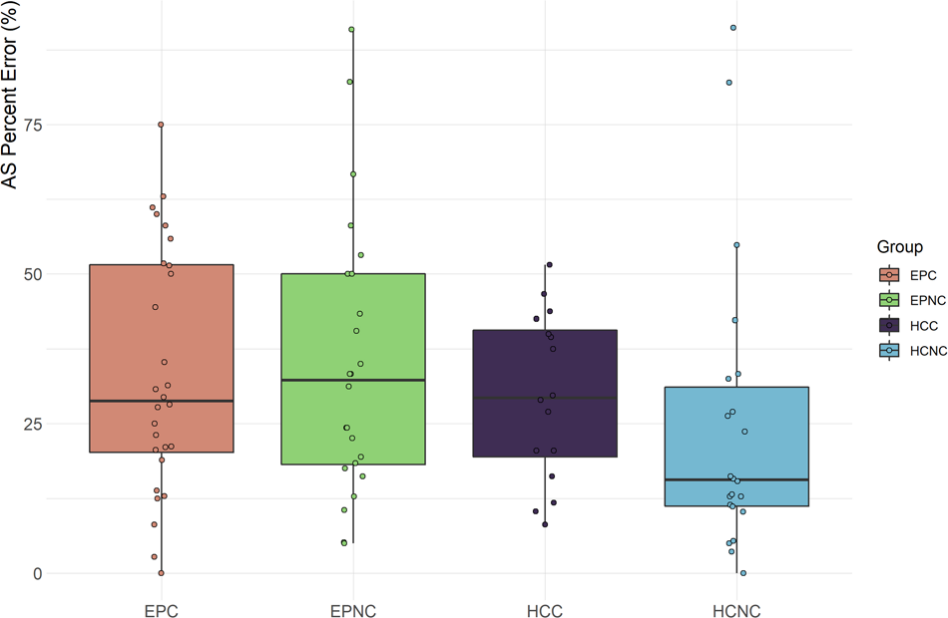
AS error × group. EPC early psychosis with cannabis use, EPNC early psychosis without cannabis use, HCC healthy controls with cannabis use, HCNC healthy controls without cannabis use, SPEM smooth pursuit eye movements. Boxplot plotted using *ggplot geom_boxplot* defaults in *R*: centre lines represent median, box hinges indicate Quartile 1 & 3, whisker 1.5x Interquartile Range from hinge or furthest data point, whichever is closer.

Antisaccade error rate by group is shown in Fig. 3 and Table 2. There was no evidence for cannabis, patient or interaction effects (*p* > 0.11) and addition of Fagerstrom and AUDIT covariates did not add information to the model (*p* = 0.69). The magnitude of difference between EPC and EPNC demonstrated minimal difference between the two groups (Hedge’s *g* = 0.08).

### Antisaccade valid trials

In an additional analysis, we found a patient effect (*χ*^2^(1) = 6.39, *p* = 0.011) such that patients made fewer valid trials compared to controls. There was no cannabis or cannabis or cannabis-patient interaction effects and AUDIT and Fagerstrom test did not add anything to the model (all ps > 0.12). See Table 2.

## Discussion

We tested two oculomotor endophenotypes of schizophrenia in patients with early psychosis and controls with and without a history of cannabis use. We provide evidence for patient and cannabis effects in SPEM velocity gain and a cannabis × patient interaction such that there is a large difference between EPNC vs EPC. In addition to the statistical analysis described we would also draw the readers’ attention to a qualitative appreciation of Fig. 2. As stimulus frequency increases, gain decreases and variance increases for all groups, as expected. The EPNC group (green bar) shows both impaired gain and increased variance compared to the other groups. In contrast, the EPC group (beige bar) behaves more in keeping with control groups. Intriguingly, we show a deficit in EPNC compared to EPC of large effect size. However, we do not find a patient or cannabis effect for AS error. Taken together, the EPNC group shows impairment in SPEM velocity gain whereas this is spared in the EPC group, but no such difference is shown in AS error rate. We also observed that patients made significantly fewer valid AS trials compared to controls, which may indicate generally poorer task performance.

These findings are of particular interest as the two patient groups were, with the exception of substance use measures, essentially indistinguishable clinically, and none of the covariates (Fagerstrom and AUDIT scores) were significantly associated with either oculomotor measure.

SPEM abnormalities are considered a trait marker unrelated to clinical state or progression of disease^20,23,25,26^ and there is good evidence from familial and genetic approaches that impairment in SPEM indexes genetic risk of schizophrenia^28,43^. One explanation of our findings, within the confines of a cross-sectional study, may be that there is less biological predisposition to psychosis for EPC compared to EPNC. This would, consistent with the decreased vulnerability hypothesis, imply that a history of cannabis use has an additive effect on psychosis liability and hence that some in this group (particularly those with the lowest predisposition to psychosis) would not have developed psychotic disorders without cannabis use, which pushed them over the psychotic threshold (Fig. 1).

An alternative explanation for our findings could be that over time chronic cannabis use may have normalised the SPEM abnormality associated with psychosis liability that may also have been present in EPC at onset of psychosis. A recent study of meditators and non-meditators showed that cultivated mindfulness is associated with fewer saccadic intrusions in SPEM. One could conceivably argue that cannabis has a similar effect for patients with psychosis through similar cognitive and attentional processes, thereby normalising SPEM^45^. It is worth noting, however, that the Kumari and colleagues findings were related to saccadic intrusions during SPEM, finding no difference in SPEM velocity between meditators and non-meditators and, therefore, does not suggest that SPEM velocity, as indexed in our study is of itself is a measure of cultivated mindfulness. A mechanism where chronic cannabis use normalises SPEM would suggest eye movements indexes state rather than trait components. In a post hoc supplementary analysis we did not find direct evidence that SPEM shows a state component in our study (post hoc correlation of SPEM with PANSS scores; *p* > 0.15). However, we accept that it is not fully possible to disentangle these explanations within the confines of a cross-sectional study. A better design to test this would be to measure eye movements before and after onset of chronic cannabis use. We note that acute THC or cannabis use has been shown to decrease rather than increase SPEM velocity gain in the previous literature^33,34^.

Our results are in line with evidence suggesting a better neuropsychological profile in cannabis-using patients at onset of psychosis compared to non-users^46^. It is however difficult to definitively address this issue as cannabis use itself may impair neuropsychological performance, and hence results have been mixed^47^. Once psychotic disorder has developed, use of cannabis indicates a poorer prognosis^8,48^. After 5 years of disorder there is evidence of detrimental effects in structural MRI measures (hippocampal, subcortical atrophy and cortical thinning) in cannabis-using patients compared to non-using patients^49^. Our results also newly identify a biomarker allowing a neurobiological distinction between these groups. Taken together, the concept of a cannabis using group with lower biological predisposition at psychosis onset but worse neurobiological indices and prognosis after long-term use indicates the utility of putatively considering cannabis-using psychosis as a distinct nosological entity. Longitudinal studies are needed to substantiate these conjectures.

It is of interest that we found differences in SPEM performance but not in relation to AS. Possible reasons for why this may be are necessarily speculative. First, although both SPEM and AS are candidate endophenotypes of schizophrenia, emerging evidence demonstrates that they are associated with differential genetic architecture^28^. It is possible, therefore, that EPC and EPNC groups differ in expression of SPEM related genes but not AS genetic expression. Exactly which genes are implicated requires further delineation. Secondly, and non-mutually exclusively it is possible that certain neurobiological substrates of the visual processing pathway are implicated in this difference which involve SPEM but not AS, such as the magnocellular system and NMDA-related mechanisms^50,51^. Specifically, the basic afferent pathway (retina–>lateral geniculate nucleus–>V1) is identical in both tasks, but differences emerge in the role of cortical regions (medial temporal area, medial superior temporal area, frontal eye fields (FEF) with modulation from the supplementary eye field and parietal cortical areas and a loop to caudate). Whilst AS involves prominent top-down control^52^, SPEM relies on target selection, motion processing, prediction and feedback processes^53^. SPEM and AS show overlapping but differential patterns of activation although definitive neurobiological substrates underpinning this distinction remain to be replicated^54,55^. Hence further work using functional MRI of eye movements within the scanner would be needed to delineate which specific areas are implicated in the observed differences between groups.

Particular strengths of this study include the use of robust and well-established biomarkers. A further strength of this study is that both patient groups were well matched across clinical parameters. This study also avoided some of the limitations of other studies comparing EPC and EPNC^49^: we accounted for tobacco or alcohol use which are more common in cannabis users^56^ and included a cannabis control group^49,57^. Finally, this study is undertaken in a real-world clinical dataset and likely to be representative of the broader clinical population.

There were limitations of this study. Groups were moderately sized, although comparable to previous eye-tracking studies, and it is possible that the inability to detect group difference in error rates in the AS task was due to low power. The cross-sectional design means it is difficult to disentangle precise casual relationships. Further work would ideally be longitudinal, charting the transition to psychotic disorders until the illness is established, or including clinical high-risk groups. We covaried for alcohol and tobacco measures, and comorbid substance use disorders were not significantly different across groups. However, in an observational setting it is not possible to completely exclude the effect of other substances of abuse, such as nicotine. We should note as discussed above that a state component to the cannabis effects cannot be precluded. Finally, whilst this study provides important new data about the EPC group, where cannabis has been heavily used as the primary drug of abuse, it is not clear whether these results are generalisable to cases of poly-substance use or patients in whom cannabis use is of mild intensity.

Notwithstanding these limitations, taken together we provide evidence for group-level differences in SPEM mean velocity gain between patients with and without a history of cannabis use. This may indicate a difference in biological diathesis to psychosis between groups. Further work is required to delineate the neurobiological substrates for this distinction.

## Method

### Sample

We undertook the Effect of Cannabis in Psychosis (EfCiP) study in 91 participants across four groups: early psychosis patients with a history of cannabis use (EPC, *n* = 28), early psychosis patients without a history (EPNC, *n* = 25), controls who had a history of cannabis use (HCC, *n* = 16) and controls who did not (HCNC, *n* = 22). All participants were aged 18–38 with comparable mean age between groups (EPC: 25.99; EPNC: 26.70; HCC: 27.11; HCNC: 27.99) and no significant difference in sex between groups (male sex: EPC: 22/28; EPNC: 16/25; HCC: 10/16; HCNC: 10/22). Participants attended a study day including standardised interview schedules, magnetic resonance imaging, eye movement testing and computer-based tasks. Each participant undertook the Structured Clinical Interview for DSM-IV (SCID-IV) screening interview and modules for affective and psychotic episodes and diagnosis and substance abuse as indicated by the screening interview to confirm group allocation.

We aimed to recruit patients in the early stages of psychosis with a first presentation of psychosis ≤5 years to secondary services. One patient under an Early Intervention in Psychosis team had a psychotic episode aged 12, 20 years previously and had been asymptomatic off-treatment until re-presenting in his 30s.

We excluded participants with psychosis due to a general medical condition (DSM IV 293), substance induced psychotic disorder other than cannabis use (DSM IV 292 excepting 292.11-12) and those intoxicated on the study day. Three out of 28 (10.7%) cannabis using patients had cannabis-induced psychotic disorder. SCID diagnoses were categorised into schizophrenia spectrum disorder (schizophrenia, schizoaffective and schizophreniform disorder) and affective disorder (bipolar affective disorder, depression with psychotic features and schizoaffective disorder). There was no difference between EPC and EPNC in the proportion of patients who had an affective psychotic disorder diagnosis (EPC 43%; EPNC 40%) or those who met criteria for schizophrenia spectrum disorders (EPC 75%; EPNC 84%).

In cannabis using groups we preferentially recruited for heavy cannabis use in both patients and controls (mean money spent in a week on cannabis EPC: £30.21; HCC £35.63). To exclude trivial or experimental use from the cannabis using groups we defined non-use as cannabis <20 times in lifetime. All EPNC and HCNC reported use either yearly or less or experimentally. All EPC had used cannabis on a monthly basis and had commenced use before presentation and 23/27 (85.1%) were using at first presentation (data not available for one person). Nine out of 28 (35.7%) EPC self-reported continuing to use cannabis and 12/28 (42.9%) tested positive for cannabis use. Patients were recruited through clinical teams from 16 NHS Trusts across England through the National Institute of Health Research (UK) Clinical Research Networks.

Controls were recruited from a list of non-psychotic volunteers as well as from an online survey thecannabissurvey.com^58^. Control participants had no history of psychotic disorder as assessed by SCID. One HCC suffered from untreated generalised anxiety disorder and one HCC had obsessive compulsive disorder 8 years ago currently in remission maintained on low dose sertraline (50 mg).

We asked participants to avoid illicit drugs 2 weeks before the study day (excluding cannabis) and avoid coffee, alcohol and cannabis on the study day. We asked participants to reduce tobacco consumption during the day but to avoid withdrawal effects did not completely disallow tobacco use and recorded time of use throughout the day. All tobacco smokers had >100 min between the last cigarette and the eye movement testing, except one participant who smoked 40 min before the task.

Ethical approval was obtained through the local research ethics committee (London-Stanmore REC 17/LO/0577). Participants signed written, informed consent before taking part in the study.

### Eye movement battery

Eye movement data were recorded from the right eye using an EyeLink 1000 (SR Research Ltd., Ottawa, Ontario, Canada) eye-tracker at a sampling rate of 1000 Hz. Stimulus display was on G90FB View Sonic Monitor with display area 360 mm (horizontal) × 270 mm (vertical), 1024 × 768 pixels, 60 Hz refresh rate. Participants were positioned with their eyes 570 mm from the monitor, on a chair adjusted for height and with the head immobilised using a comfortable chin rest with forehead restraint.

The eye movement battery was written in ExperimentBuilder (SR Research Ltd.). The visual stimulus consisted of a white circle (0.3° diameter) on a black background with movement restricted to the horizontal plane. The smooth pursuit stimulus used a sinusoidal waveform at three frequencies (0.2, 0.4 and 0.6 Hz) across a horizontal range of ±13°. Instructions were to follow the stimulus as closely as possible without moving the head. Following smooth pursuit, participants also underwent assessment of antisaccades. Following two blocks of three practice trials each, participants performed 40 antisaccade trials using a horizontal step task with an equal number of left (−13°) and right (+13°) stimuli in randomised order. In each trial, the stimulus (a white circle of 0.3° diameter) was shown in the central position (0°) for 1000–1900ms before it stepped, without gap or overlap, to the right or left where it remained for 1000 ms. Instructions were to not look at the peripheral stimulus, but instead to look at the exact mirror image location as soon and accurately as possible. Data recording was preceded by a horizontal and vertical 5-point calibration.

Eye movement data quality was first checked using DataViewer (SR Research Ltd.), blinded to participant status. Then, we used in-house Matlab (The Mathworks, Natick, MA, USA) scripts^59–61^ to calculate the following primary dependent variables:

(1) SPEM velocity gain = eye velocity/stimulus velocity for the middle 50% of each half-cycle, with saccades and blinks having been excluded; calculated separately for each frequency (0.2, 0.4 and 0.6 Hz).

(2) AS error rate = number of direction errors/number of valid trials, expressed as percentage.

For an AS trial to be valid (i) the saccade needed to end before the peripheral stimulus disappeared; (ii) it must have been preceded by a fixation of at least 100 ms before stimulus onset without blinks; (iii) the saccade must have commenced from the central position (±100 pixels); (iv) the latency must have been at least 80 ms; and (v) the amplitude had to be at least 1°. A directionally correct antisaccade was defined as the first valid saccade in a trial if it occurred in opposite direction of the stimulus. A direction error was defined as the first valid saccade in a trial but in the direction of the stimulus.

Whilst the number of valid saccades was not a primary outcome, at a reviewer’s request we subsequently considered this variable in a supplementary analysis.

### Clinical measures

All psychiatric diagnoses as assessed by the SCID were made by one of two trained raters and experienced clinicians (clinical psychiatry experience >6 years each). Data collected included Global Assessment of Functioning (GAF), Global Functioning and Social Functioning Scales (GFS, SFS)^62^ and Positive and Negative Syndrome Scale (PANSS) (3 independent raters, intra-class coefficient 0.87). National Assessment of Reading Test (NART) was used to index full-scale intelligence quotient using a restandardised calculation in British adults^63^. One participant with mild learning disability was assigned a score of 65. Participants were administered the Fagerstrom test for Nicotine Dependence^64^, the Alcohol Use Disorders Identification Test (AUDIT), the Severity Dependence Scale (SDS)^65^ in relation to the last month’s cannabis use, the TimeLine FollowBack questionnaire^66^ and a modified version of the Cannabis Experiences Questionnaire^67,68^. Antipsychotic medication exposure in EPC and EPNC was estimated as chlorpromazine equivalents, calculated from the Maudsley Guidelines 12th Edition, or where not available therein, from Gardener et al.^69,70^.

### Statistical analysis

Demographic and clinical variables were compared across groups using ANOVA and chi squared tests as appropriate using SPSS version 25. Because the primary comparison of interest was EPC vs EPNC these differences were also specifically tested using *t*-tests and *χ*^2^ tests. *P*-value threshold was set at 0.05 (see Table 1).

Distributions of eye movement measures were inspected for normality and transformed as appropriate. Specifically, SPEM measures and AS valid trials were logarithmically transformed to correct positive skew and AS error rate was square root transformed to correct negative skew. We constructed full linear models for eye movements data as a function of patient and cannabis user status as described below. For mixed models we used the lme4 package and for fixed factor models we used the linear regression lm function and lmtest function in R 3.6.3 as described below.

For SPEM gain, we fit a mixed model using the lme4 function in R^71^. This class of models allows for the minimisation of assumptions in repeated measures designs^72^. We constructed a model with patient status and cannabis user status as fixed factors and gain at the three frequencies as random factors (intercept). In order to determine whether the factors in the model were significant we also constructed alternative models (patient status only, cannabis status only) and an interaction model (patient status × cannabis user). To determine the significance of each factor, models were compared using a likelihood ratio test, comparing nested models. *P*-value threshold was set at *p* < 0.05. This allowed us to determine a statistically optimal model.

For antisaccades errors and valid trials, a model was fit using patient status and cannabis user status as fixed factors for the full model using the *lm* function. Alternative models (patient status only, cannabis status only, interaction) were generated and compared to the full model via likelihood ratio testing. *P*-value threshold was set at *p* < 0.05.

In both SPEM gain and antisaccade error and valid trial models we also added covariates (AUDIT and Fagerstrom to index alcohol and smoking status respectively) as fixed factors to determine if the covariates were informative in the model.

Finally, to specifically determine the magnitude of difference between EPC and EPNC in both SPEM and AS we calculated Hedge’s g.

## Data Availability

The dataset generated during and/or analysed during the current study are available from the corresponding author on reasonable request.

## Code availability

The code generated for extraction and analysis of the data during the current study are available on reasonable request.
